# Hip joint replacement surgery for idiopathic osteoarthritis aggregates in families

**DOI:** 10.1186/ar1878

**Published:** 2006-01-03

**Authors:** H Bukulmez, AL Matthews, CM Sullivan, C Chen, MJ Kraay, RC Elston, RW Moskowitz, VM Goldberg, ML Warman

**Affiliations:** 1 Department of Genetics and Center for Human Genetics, Case Western Reserve University and University Hospitals of Cleveland, Cleveland, Ohio, USA; 2 Department of Epidemiology and Biostatistics, Case Western Reserve University and University Hospitals of Cleveland, Cleveland, Ohio, USA; 3 Department of Pediatrics at Metro Health Medical Center, Case Western Reserve University and University Hospitals of Cleveland, Cleveland, Ohio, USA; 4 Center for Human Genetics, Case Western Reserve University and University Hospitals of Cleveland, Cleveland, Ohio, USA; 5 Department of Orthopaedics, Case Western Reserve University and University Hospitals of Cleveland, Cleveland, Ohio, USA; 6 Department of Medicine, Arthritis Translational Research Program, Case Western Reserve University and University Hospitals of Cleveland, Cleveland, Ohio, USA

## Abstract

In order to determine whether there is a genetic component to hip or knee joint failure due to idiopathic osteoarthritis (OA), we invited patients (probands) undergoing hip or knee arthroplasty for management of idiopathic OA to provide detailed family histories regarding the prevalence of idiopathic OA requiring joint replacement in their siblings. We also invited their spouses to provide detailed family histories about their siblings to serve as a control group. In the probands, we confirmed the diagnosis of idiopathic OA using American College of Rheumatology criteria. The cohorts included the siblings of 635 probands undergoing total hip replacement, the siblings of 486 probands undergoing total knee replacement, and the siblings of 787 spouses. We compared the prevalence of arthroplasty for idiopathic OA among the siblings of the probands with that among the siblings of the spouses, and we used logistic regression to identify independent risk factors for hip and knee arthroplasty in the siblings. Familial aggregation for hip arthroplasty, but not for knee arthroplasty, was observed after controlling for age and sex, suggesting a genetic contribution to end-stage hip OA but not to end-stage knee OA. We conclude that attempts to identify genes that predispose to idiopathic OA resulting in joint failure are more likely to be successful in patients with hip OA than in those with knee OA.

## Introduction

Osteoarthritis (OA) is a multifactorial disease. Environmental, hormonal, obesity, mechanical, and genetic factors have been implicated in its onset and progression [1]. OA is clinically heterogeneous because it can affect large or small joints, can be monoarticular or polyarticular, and can be associated with subtle or obvious physical and/or radiographic changes. OA is also heterogeneous with respect to the histologic, biochemical, and molecular changes observed in bone, cartilage, and synovial cells and matrices.

OA causes substantial morbidity and disability, as well as economic costs [2]. Consequently, substantial efforts have been made to identify factors that can affect the incidence and progression of OA. Mendelian genetic disorders that have precocious joint failure as a component feature are an uncommon cause of OA [3], but their existence suggests that other genetic variants may contribute to common forms. Further evidence supporting a genetic contribution to common OA derives from studies looking at aggregation of OA within families [4-10] and within ethnic/geographic groups [11-14], and in twins [15-17].

In light of the evidence supporting a genetic contribution to common OA, investigators have sought to identify genetic variants, or chromosomal regions predicted to contain variants, that contribute to common forms of OA. Many variants have been suggested as risk factors for common OA based on association studies [18-25]. Similarly, genome-wide searches using relatives concordant for OA have suggested several chromosomal regions as containing susceptibility loci [14,26-29]. Importantly, statistical evidence in favor of chromosomal regions (or genes) increased when the original patient cohorts were stratified into subgroups based on sex, affected joint site, number of affected joints, and severity of joint disease, such as having undergone joint replacement [26,27,30-32]. Chromosome 4 and 16 linkages are specific to female affected families with hip OA who had undergone total hip replacement (THR) [30]; chromosome 2q linkage was found in distal interphalangial joint OA [33], knee OA [34] and hip OA [35]; and 11q linkage is specific to female OA [36] and hip OA [37]. When the linkage signals are analyzed after the OA affected individuals are stratified based on knee/hip joint arthritis and sex, it is observed that most of the linkages are consistent for hip OA [32,35,38,39], but they are not consistent for knee OA or sex specific.

However, to date, few putative disease-predisposing variants identified by one genetic method have been confirmed using another genetic method. These include secreted frizzled related protein 3 for hip OA [35], matrillin-3 for hand OA [28], and a locus on chromosome 6p for hip OA in women [40]. These recent data are intriguing, but their replication in other cohorts is required to confirm their causality and to distinguish them from positive associations due to type I error (for instance, a false-positive result).

In the present study, using joint replacement surgery for idiopathic OA as a qualitative measure of joint failure, we sought to determine how hip joint failure and knee joint failure cluster in families. Finding familial aggregation for one or both of these traits would support their usefulness for finding and testing candidate genes. Conversely, failure to find familial aggregation would suggest that these traits are not useful in genetic studies. As an appropriate control group for comparison, we chose to use the siblings of the probands' spouses. Other studies have used spouses as control individuals [6,8] presumably because they are more likely to be matched for ethnic/geographic ancestry and socioeconomic status. We used siblings of spouses, which comparably match for ethnic/geographic ancestry and socioeconomic background but they better enable us to delineate confounding variables such as sex and environment [41].

Herein, we report that hip arthroplasty aggregates in families independent of potential confounders such as age and sex. In contrast, knee arthroplasty does not aggregate in families independent from these factors. These data suggest that stratifying patient cohorts based on the presence of hip arthroplasty is appropriate for genetic studies, whereas stratifying based on the presence of knee arthroplasty is not.

## Materials and methods

The study was approved by the Institutional Review Board at University Hospitals of Cleveland. All participating probands signed an informed consent. All spouses and affected siblings who were contacted to confirm their site of and age at arthroplasty gave written or verbal consent.

### Patient ascertainment/recruitment

Probands and their spouses were invited to participate in the study by the two surgeons (VMG and MJK), who performed more than 85% of the hip and knee arthroplasties for OA at the University Hospitals of Cleveland. The period of recruitment was the years 1997–2001. The participation rate in the study was in excess of 90%. The probands had either recently undergone or were scheduled to undergo a hip or knee arthroplasty to treat severe disability caused by idiopathic OA. Idiopathic OA was defined by American College of Rheumatology criteria [42,43]. In order to validate the diagnosis, radiographic, laboratory, and physical examination findings were reviewed. Siblings who had joint replacements were interviewed to ascertain the reason for their joint replacement. Probands or affected siblings with skeletal dysplasia, congenital malformations, history of joint trauma, or whose arthroplasty followed a fracture were considered unaffected. Because OA of one major joint may predispose to OA in a second joint [44], we only included as probands (and affected siblings) those who were undergoing an arthroplasty of either the hip or the knee joint. The group of all siblings of probands who had undergone THR is referred to as the 'THR proband sibling' cohort. The group of all siblings of probands who had undergone total knee replacement (TKR) is referred to as the 'TKR proband sibling' cohort. The 'spouse sibling' cohort comprises all siblings of the TKR and THR probands' spouses. The spouse sibling cohort did not share the same environment and comprised genetically unrelated individuals, because they were siblings of the spouses and not of the probands.

### Data collection

Information collected from each proband, their siblings, spouses, and siblings of spouses included date of birth, sex, ethnic background, education status, occupation, other joints affected with symptomatic OA, age at joint replacement(s), and site(s) of joint replacement. Only probands older than 40 years were included in the study. Family histories were collected from each proband and spouse specifically addressing whether their siblings had symptomatic OA or had undergone an arthroplasty for idiopathic OA. Age, sex, joints affected with symptomatic OA, and age(s) and site(s) of arthroplasty for idiopathic OA were recorded for each sibling who survived beyond age 40 years. Height and weight (to calculate body mass index [BMI]) were recorded from a subset of siblings (3%) who had undergone an arthroplasty for idiopathic OA. Arthroplasties in siblings that were attributed to other causes (for instance, trauma, fracture, rheumatoid arthritis) were not included when the data were analyzed.

### Statistical analyses

Logistic regression, Pearson correlation, and descriptive statistics (means and standard deviations) were calculated using the SAS (v8.0) software package (SAS version 8; SAS Institute Inc., Cary, NC, USA). To allow for sibling correlations in the logistic regression, we used a multivariate logistic regression model that includes first order correlations, as implemented in the SEGREG program of the SAGE package (SAGE 4.3 [2004] Statistical Analysis for Genetic Epidemiology [45]). Contingency table χ^2 ^analysis was used to compare risks for joint arthroplasty among siblings with OA when the proband had unilateral versus bilateral arthroplasty. The Student's *t *test was used to compare the means of age and BMI. Logistic regression analyses considered age, sex, personal history of prior joint arthroplasty, and family history of arthroplasty in a sibling as potential risk factors. We aimed to obtain a valid estimate of the risk in siblings of individuals who had undergone arthroplasty surgery (secondary to severe OA) on their knees or hips as compared with their spouses' siblings as control individuals. We defined a 'group' variable that is dichotomous and represents the siblings of hip (or knee) replaced individuals as one group and siblings of the spouses as another group. We initially allowed for main effects, and two-way and three-way interactions between variables. Then, we successively eliminated the nonsignificant interactions and main effects for which significance was *P *> 0.1. Thus, a model containing main effects (hip or knee replacement) and all interactions of the variables such as age at surgery, age, sex, and the group that the individuals belong to were tested against models with variables removed one by one, eliminating first three-way interactions, then two-way interactions, and finally nonsignificant main effects.

## Results

### Family data

#### Hip arthroplasty probands and the THR proband sibling cohort

We invited 763 individuals who were scheduled for hip arthroplasty to participate in the study, and 710 individuals agreed to participate. In order to evaluate the familial aggregation of hip arthroplasty independent of replacements involving other joints, we excluded from this cohort 75 individuals (10.5%) who were scheduled for hip arthroplasty and had either a history of prior knee arthroplasty or were scheduled for concurrent hip and knee replacement. Characteristics of the remaining 635 hip-only probands are summarized in Table 1. There were more females than males (female/male ratio = 1.57), with no statistically significant difference in their mean ages and BMIs. These probands had a total of 1533 siblings surviving beyond 40 years of age (2.3 siblings/proband). There were nearly equal numbers of female and male siblings (female/male ratio = 1.02), and the mean age of the siblings did not differ from the mean age of the probands (*P *> 0.05). Fifty-seven siblings (3.7%) had also undergone hip arthroplasty, 22 (1.4%) had undergone knee arthroplasty, and eight (0.5%) had undergone hip and knee arthroplasty for idiopathic OA. The ratio of female to male siblings who had hip replacement was 1.1, and the mean age of siblings who had hip replacement was about 5 years older at the time of surgery than the mean ages of the probands and the unaffected siblings (*P *< 0.001 for both comparisons). The ratio of female to male siblings who had undergone knee replacement was 0.7 and the mean age at the time of surgery was about 6 years older than the mean ages of the probands and the unaffected siblings (*P *= 0.001).

#### Knee arthroplasty probands and the TKR proband sibling cohort

We invited 601 individuals who were scheduled for knee arthroplasty to participate in the study and 570 individuals agreed to participate. However, in order to evaluate the familial aggregation of knee arthroplasty independent of replacements involving other joints, we excluded from this cohort 84 individuals (14.7%) who had either a history of previous hip arthroplasty or who were scheduled for concurrent hip and knee replacement. Characteristics of the knee-only probands are also summarized in Table 1. There were more females than males (female/male ratio = 2.48), with no statistically significant differences in their mean ages or BMIs. These 486 probands had a total of 1208 siblings who survived above 40 years of age (2.8 siblings/proband), there were nearly equal numbers of female and male siblings (female/male ratio = 1.03), and the mean age of the siblings was about 2.5 years younger than the mean age of the probands (*P *= 0.001). Forty-six (3.8%) of the siblings had also undergone knee arthroplasty, 24 (1.9%) had undergone hip arthroplasty, and five (0.4%) had undergone hip and knee arthroplasty for idiopathic OA. The ratio of female to male siblings who had undergone knee arthroplasty was 1.4 and the mean age of siblings who had undergone knee arthroplasty was about 2 years older than that of the probands (*P *= 0.001) and about 6 years older than the mean age of the unaffected siblings (*P *< 0.001). The ratio of female to male siblings who had undergone hip replacement was 1.9 and their mean age was about 6 years older than the mean age of the unaffected siblings (*P *< 0.001) and about 3 years older than the probands (*P *= 0.12).

#### Spouses and the spouse sibling cohort

Spouses of both hip and knee replaced individuals were invited to participate in the study. A total of 787 spouses participated, which represented more than 95% of all eligible spouses. The spouses had a total of 1,900 siblings surviving beyond 40 years of age (2.4 siblings/spouse). The characteristics of the siblings are summarized in Table 1. There were equal numbers of female and male siblings (female/male ratio = 1.03). Twenty-five (1.3%) of the siblings had undergone knee arthroplasty, 24 (1.3%) had undergone hip arthroplasty, and five (0.3 %) had undergone hip and knee arthroplasty for idiopathic OA. The ratio of female to male siblings with knee arthroplasty was 2, and the mean age of affected female siblings was about 7 years older than the mean ages of the affected males and the unaffected siblings (*P *< 0.001). The ratio of female to male siblings with hip arthroplasty was 1.4 and the mean age of the affected individuals was about 4 years older than the mean age of the unaffected siblings (*P *< 0.001).

### Aggregation of arthroplasty within the THR proband sibling cohort

The prevalence of hip arthroplasty in the THR proband siblings was 3.7%, which was significantly greater than the 1.3% prevalence in the control (spouse sibling) cohort (odds ratio = 2.8, 95% confidence interval = 1.8–4.8). In contrast, there was virtually no difference in the prevalence of knee arthroplasty (1.4% versus 1.3%; odds ratio 1.05, 95% confidence interval = 0.59–1.86) between these cohorts. Logistic regression analysis (Table 2) using the SAS (ignoring sibling correlations) and SEGREG (allowing for sibling correlations) programs identified age (*P *< 0.001) and having a sibling with a THR (*P *< 0.001) as independent risk factors for hip arthroplasty in the THR proband siblings. However, having a sibling who had undergone TKR did not significantly increase the risk for hip arthroplasty (*P *> 0.4). These findings support there being a genetic contribution to hip arthroplasty risk but not to knee arthroplasty risk (Wald χ^2^: 20.18; *P *< 0.001) among siblings of THR probands. In addition, logistic regression did not identify 'sex' as an independent risk factor for hip arthroplasty (Table 2).

### Aggregation of arthroplasty within the TKR proband sibling cohort

The prevalence of knee arthroplasty in the TKR proband siblings was increased compared with the prevalence in the control (spouse sibling) cohort (3.8% versus 1.3%; odds ratio = 2.9, 95% confidence interval = 1.77–4.73). Logistic regression analysis (Table 3) identified age (*P *< 0.001), prior personal history of hip replacement (*P *< 0.001), and sex (*P *= 0.008) as independent risk factors for knee replacement in the TKR proband siblings. Additionally, an interaction between female sex and older age increased the risk for having a knee arthroplasty (*P *= 0.001) in this group. Importantly, although the crude odds ratio indicated increased risk for TKR in TKR proband siblings, logistic regression did not identify having a sibling with a TKR to be an independent risk factor for hip or knee arthroplasty in the TKR proband siblings (*P *> 0.5). These findings do not support there being a genetic contribution to the risk for knee arthroplasty. Increased risk for TKR among siblings of TKR probands observed by crude odds testing might be explained by prior personal experience of successful hip arthroplasty, female sex, or old age. Because the crude odds ratio calculation does not control for all of these confounding factors, it cannot indicate whether there is residual familial aggregation of knee arthroplasty. Logistic regression analysis accounted for these confounding factors.

### Correlation of body mass index with the risk of knee or hip arthroplasty

We did not collect BMI data from all proband siblings, spouses, or the spouses' siblings, and so we could not include BMI in the logistic regression analyses. To determine whether BMI correlated with the risk for hip arthroplasty, we recorded height and weight data from approximately 3% of the siblings (focusing on families with living affected siblings) and calculated Pearson correlation coefficients between the hip probands and their siblings, and between knee probands and their siblings. We conducted the correlation analysis controlling for age.

When the THR probands and their siblings were analyzed, we found that male siblings' BMI did not correlate with hip arthroplasty (*P *= 0.7) but the trend was toward correlation with knee arthroplasty (*P *= 0.08). In female siblings there was a correlation between BMI and hip arthroplasty (*P *= 0.01), but interestingly the risk for hip arthroplasty correlated with low BMI rather than with high BMI. Knee arthroplasty in affected female siblings correlated with increased BMI (*P *= 0.02). Surprisingly, when TKR probands and their siblings were analyzed, there were no significant correlations between knee arthroplasty and BMI, or between hip arthroplasty and BMI, in either sex.

### Risk for arthroplasty in siblings of probands with unilateral versus bilateral hip or knee arthroplasty

In many diseases, such as cancer, individuals for whom there is a strong heritable contribution often have multiple affected sites. Therefore, we separated hip arthroplasty and knee arthroplasty probands on the basis of their having unilateral or bilateral replacements. Then we determined whether having bilateral joint replacement increased the prevalence of arthroplasty in their siblings (Tables 4 and 5). The mean ages between TKR probands did not differ. Siblings of probands with bilateral hip replacements had greater rates of arthroplasty than did siblings of probands with unilateral hip replacements (Table 4; χ^2 ^[degrees of freedom (df)] = 23.6 [3 df]; *P *< 0.001). In contrast, siblings of probands with bilateral knee replacements did not have significantly greater rates of arthroplasty than did siblings of probands with unilateral knee replacements (Table 5; χ^2 ^[df] = 1.39 [3 df]; *P *> 0.5). These data are consistent with there being a genetic contribution to end-stage hip OA but not to end-stage knee OA.

## Discussion

In order to find genes that confer risk for phenotypic traits, these traits must *a priori *be under genetic influence and hence familial. We sought to determine whether the phenotypic traits of having a hip or knee arthroplasty for idiopathic OA were under genetic influence by comparing the prevalence of arthroplasty in siblings of affected individuals with that in control individuals. Prior studies have compared rates of OA between probands and spouses [6,8] or between probands and population controls [5,7,9,46,47]. The former comparison may be confounded because spouses share common environmental factors and are opposite in sex. The latter comparison may be confounded because population controls may have different ethnic and socioeconomic backgrounds than the probands. In the present study we chose siblings of the probands' spouses as the control group, assuming that assortive mating for ethnicity and socioeconomic background would be more prevalent.

We found that hip arthroplasty is significantly increased in the siblings of THR probands when compared with the siblings of spouses, even after controlling for age and sex. Previous reports [39,48-50] have suggested that increased BMI is associated with hip and knee arthroplasty. Therefore, assuming that increased BMI could be a cause of the familial aggregation, we also collected BMI data from some of the study participants to determine the correlation of BMI with knee and hip arthroplasty. There were no significant increases in BMI with hip arthroplasty, whereas knee arthroplasty did exhibit an increase. These data lend further support to the contention that genetic factors contribute to the aggregation of end-stage hip OA but not knee OA. However, the data cannot preclude other shared factors (vocation, exercise habits) as being responsible for the familial aggregation rather than shared genes. In contrast, we found that knee arthroplasty was not increased among the siblings of TKR probands after controlling for age and sex. This finding argues against either genetic or shared environmental contributions to end-stage knee OA in families.

Our study agrees with prior studies that found increased familial aggregation for hip arthroplasty [6,7,14] and lends support to studies that found increased aggregation of hip OA defined by other measures [5,13]. Although our results do not support an earlier study that suggested increased familial aggregation for knee arthroplasty [6], they are consistent with those of another study [39] that also did not find familial aggregation for knee OA. It is important to emphasize that because this study recruited patients who underwent joint replacement surgery, it only addresses the role of genetics in severe forms of knee and hip OA, because we looked at arthroplasty rates and not at other measures of arthritis employed in prior studies [8,10].

The most important outcome of our study is that, when considering arthroplasty as a phenotypic trait, only hip arthroplasty is likely to be under genetic influence. Few gene association studies have been performed that specifically looked at genetic variants as risk factors for joint arthroplasty [35,38]. Most association studies compared allele rates between cases and controls (defined by the presence and absence of clinical or radiographic arthritis changes) or between severely and mildly affected individuals (defined by Kellgren-Lawrence or other OA scales) [18-20,23,51]. Therefore, our finding does not allow us to comment definitively on the conclusions of these studies. In contrast, several studies of excess allele sharing among concordant sibling pairs utilized participants with arthroplasty for idiopathic OA as the phenotypic trait; these studies found increased support for linkage between chromosomal regions and arthroplasty after stratification of the data by site of arthroplasty and sex [27,32,52,53]. Furthermore, the linkage signals increased when female or male hip affected sibling pairs were analyzed separately [30,31,54]. Although stratifying cohorts into smaller groups may increase the ability to detect linkage by decreasing heterogeneity, it can also lead to type I errors because of the multiple additional hypotheses being tested [55]. Our findings suggest that these investigators' stratification based upon hip replacement is appropriate. Although our data do not support stratifying hip replacement cohorts based on sex, our study design might not have been able to detect sex-dependent familial aggregation. Hawker and coworkers [56] found that, despite having a higher prevalence of severe arthritis (odds ratio = 1.76; *P *= 0.001), women significantly under-utilize arthroplasty as a treatment compared with men. Because our cohorts were ascertained only when probands underwent arthroplasty, our results may show less risk for female familial OA.

In the hip sibling cohort, in addition to family history, we identified age as an independent risk factor. This agrees with other studies [57] and the common observation that the prevalence of OA increases as humans age [58]. In the knee sibling cohort, we identified age and sex as independent risk factors, which also agrees with previous studies [57-59].

We speculate that shared genes account for familial aggregation of hip arthroplasty. However, other factors, such as access to medical care and communication between family members who may have experienced improved quality of life following arthroplasty, could also contribute to this clustering. These two explanations seem unlikely to account for the increased rate of hip replacement in our study because siblings did not have increased rates of knee replacement compared with control individuals. One would expect overall increases in the rate of arthroplasty if health insurance, access to health care, and 'word of mouth' were important factors in influencing a sibling's decision to undergo arthroplasty.

Increased BMI has been reported to correlate consistently with knee OA, although correlations with hip OA have yielded inconsistent results [50]. In this study correlations between BMI and arthroplasty were inconsistent. This may relate to the fact that the mean BMIs in our probands were in the overweight to mildly obese range (25.8–30.2 kg/m^2^) and that we collected BMI data from only a small subset of the siblings. Accordingly, skewed BMI distributions and small sample size in our cohort might have lessened our ability to demonstrate a clear correlation between BMI and arthroplasty risk. However, we did find a significant correlation between BMI and knee arthroplasty in the female siblings of hip OA probands and a trend toward significance in the male siblings, which supports prior studies describing BMI as a fundamental risk factor for knee joint failure [60,61].

In the TKR proband sibling cohort, a prior history of hip replacement was an independent risk factor for having a knee arthroplasty. Several explanations may account for this result. First, these individuals may have had severe forms of OA that affected several joints concurrently. To address the question of whether a more severe form of OA could account for this observation, we stratified probands based on their having unilateral versus bilateral arthroplasty. Arthroplasty rates were higher in siblings of probands who had bilateral hip replacement but not bilateral knee replacement. Because we excluded probands having both a hip and a knee arthroplasty from this study, we cannot comment on whether a more generalized form of OA that causes end-stage OA in multiple joints will also cluster in families. Second, arthritis in the second joint may have arisen as a consequence of disability caused by arthritis in the first joint [44]. Third, the threshold for having a second arthroplasty may be reduced in individuals who had a satisfactory result from their first replacement.

Among the strengths of our study is that it ascertained probands who underwent only hip arthroplasty or only knee arthroplasty, and recruited a control group that did not share a common household but was comparable in terms of age and ethnicity. We also applied a statistical analysis (SEGREG) that allowed for sibling correlations. Prior association studies of hip and knee OA compared affected and unaffected family members or siblings of affected individuals with the general population. These types of studies are more difficult to control for shared environment in families, population stratification, and/or site(s) of joint involvement.

The limitations of our study are that we focused solely on joint arthroplasty as a qualitative trait. Although this was a cost-effective way to identify a population over the age of 40 years that is affected with end-stage, debilitating OA, it missed the larger proportion of the population that is affected with less severe forms of the disease. Therefore, our study design was unable to determine whether other characteristics of OA, such as age of onset, degree of pain, or rate of progression to joint failure, were genetically influenced. Although we were able to confirm the diagnosis of idiopathic OA in the probands by reviewing radiographs and performing physical examinations, we were unable to do this for all of their or their spouses' affected siblings. However, telephone interviews with the affected siblings supported the accuracy of the probands' and their spouses' recollections when describing the age, site, and reason for arthroplasty in the siblings. Finally, our study cohorts comprised US residents from northeast Ohio who have diverse ethnic/geographic ancestries, and findings may be different in other populations.

## Conclusion

Hip arthroplasty for idiopathic OA clusters in families, but knee arthroplasty does not. Therefore, attempts to identify genes that predispose to idiopathic OA resulting in joint failure are more likely to be successful in patients with hip OA than in those with knee OA.

## Abbreviations

BMI = body mass index; df = degrees of freedom; OA = osteoarthritis; THR = total hip replacement; TKR = total knee replacement.

## Competing interests

The authors declare that they have no competing interests.

## Authors' contributions

All authors participated in the writing of the manuscript. HB, CC, and RCE performed the statistical analyses. VMG, MJK, RWM, ALM, CMS, and MLW participated in the initial design of the study, recruiting participants, obtaining informed consent and confirming the diagnosis of idiopathic OA.

## Figures and Tables

**Table 1:** Proband, spouse, and sibling characteristics

Participants	Number	Sex (female/male ratio)	Mean age (female/male [SD])	Mean BMI (female/male [SD])
THR probands	635	384/251 (1.5)	69.4 (9.2)/66.9 (11.4)	28.8 (6.3)/28.5 (3.6)*
TKR probands	486	344/142 (2.5)	69.8 (9.1)/71.1 (8.1)	30.3 (6.9)/28.5 (2.8)*
Spouses	787	290/497 (0.6)	67.2 (9.0)/69.6 (9.1)	-
Siblings combined				
THR proband siblings	1,533	775/758 (1.0)	67.1 (12.2)/66.1 (11.5)	-
TKR proband siblings	1,208	639/569 (1.1)	67.3 (11.2)/65.9 (26.5)	-
Spouse siblings	1,900	968/932 (1.0)	68.2 (11.4)/66.22 (11.17)	-
Siblings with hip replacement				
THR proband siblings	57 (3.7%)	30/27 (1.1)	72.4 (10.2)/72.2 (8.5)	-
TKR proband siblings	24 (1.9%)	15/9 (1.6)	72.2 (9.6)/74.5 (12.3)	-
Spouse siblings	24 (1.3%)	14/10 (1.4)	74.2 (9.07)/73.7 (9.47)	-
Siblings with knee replacement				
THR proband siblings	22 (1.4%)	9/13 (0.7)	76.55 (7.03)/73.69 (8.3)	-
TKR proband siblings	46 (3.8%)	27/19 (1.4)	72.7 (9.3)/72.3 (10.7)	-
Spouse siblings	25 (1.3%)	18/9 (2.0)	75.4 (8.5)/68.5 (10.7)	-
Siblings with no replacement				
THR proband siblings	1,454	730/724 (1.0)	67.7 (11.4)/66.3 (26)	-
TKR proband siblings	1,138	597/541 (1.1)	66.7 (11.4)/65.5 (26)	-
Spouse siblings	1,851	937/914 (1.0)	68.1 (11.4)/66.1 (11.2)	-

Demographic data for the THR probands and their siblings (THR proband siblings), the TKR probands and their siblings (TKR proband siblings), and the probands' spouses and the spouses' siblings (spouse siblings). Mean age and BMI values are provided separately for females and males, as are the standard deviations. *BMI was derived from 96 randomly chosen hip arthroplasty probands and 73 randomly chosen knee arthroplasty probands. BMI, body mass index; SD, standard deviation; THR, total hip replacement; TKR, total knee replacement.

**Table 2:** Risk factors for hip arthroplasty modelled by logistic regression in the THR proband sibling cohort

Factor	*P *value	
	
	Ignoring sibling correlations	Allowing sibling correlations
Age	<0.001*	<0.001*
Sibling with hip arthroplasty	<0.001*	<0.001*
Personal prior history of knee arthroplasty	0.39	0.46
Female sex	0.33	0.30

*Statistically significant findings. THR, total hip replacement.

**Table 3:** Risk factors for knee arthroplasty modelled by logistic regression in the TKR proband sibling cohort.

Factor	*P *values	
	
	Ignoring sibling correlations	Allowing sibling correlations
Age	<0.001*	<0.001*
Sibling with knee arthroplasty	>0.5	>0.5
Prior personal history of hip replacement	<0.001*	<0.001*
Female sex	0.009*	0.008*

*Statistically significant findings. TKR, total knee replacement.

**Table 4:** The prevalence of arthroplasty in THR proband siblings stratified by unilateral versus bilateral replacement

Probands (*n *= 635; mean age in females/males, years [SD])	THR proband siblings (*n *= 1533)			
	
	UHR	BHR	Unaffected	Total
UHR (*n *= 463; 69.3 [9]/67.2 [11.3])	18 (1.6%)	15 (1.3%)	1,079 (96.1%)	1,122
BHR (*n *= 172; 69.7 [9.4]/65.9 [11.3])	17 (4.1%)	7 (1.7%)	375 (91.1%)	411
Total	35	22	1,454	1,533

χ^2 ^(3 degrees of freedom) = 23.6; *P *< 0.001. BHR, bilateral hip replacement; SD, standard deviation; THR, hip replacement; UHR, unilateral hip replacement.

**Table 5:** The prevalence of arthroplasty in TKR proband siblings stratified by unilateral versus bilateral joint replacement

Probands (n = 486; mean age in females/males, years [SD])	TKR proband siblings (*n *= 1208)			
	
	UKR	BKR	Unaffected	Total
UKR (n = 300; 70.1 [7.2]/70.5 [6.7])	16 (2.0%)	8 (1.0%)	743 (94.1%)	790
BKR (n = 186; 69.8 [8.3]/69.4 [9.6])	12 (2.9%)	4(0.9%)	392 (93.8%)	418
Total	28	12	1,133	1,208

χ^2 ^(3 degrees of freedom) = 1.39, *P *> 0.5. BKR, bilateral knee replacement; SD, standard deviation; TKR, total knee replacement; UKR, unilateral knee replacement.
